# Performance Evaluation of Four Qualitative RT-PCR Assays for the Detection of Severe Acute Respiratory Syndrome Coronavirus 2 (SARS-CoV-2)

**DOI:** 10.1128/spectrum.03716-22

**Published:** 2023-02-28

**Authors:** Riffat Munir, Lesley Erica Scott, Lara Dominique Noble, Kim Steegen, Lucia Hans, Wendy Susan Stevens

**Affiliations:** a WITS Diagnostic Innovation Hub, Faculty of Health Sciences, University of the Witwatersrand, Johannesburg, South Africa; b Department of Molecular Medicine and Haematology, School of Pathology, Faculty of Health Science, University of the Witwatersrand, Johannesburg, South Africa; c National Priority Program of the National Health Laboratory Services (NHLS), Johannesburg, South Africa; Oklahoma State University

**Keywords:** coronavirus, COVID-19, *in vitro* diagnostics, RT-PCR, SARS-CoV-2, analytical performance

## Abstract

Severe acute respiratory syndrome coronavirus 2 (SARS-CoV-2), emerged in late 2019, and its rapid spread around the globe led the World Health Organization to declare it a pandemic. Laboratory diagnostics provide important information to help control virus transmission, and molecular nucleic acid amplification tests have been recognized as the gold standard for the direct detection of viral genetic material. The main aim of this study was to independently evaluate the analytical performance of four molecular assays that were designed for the detection of SARS-CoV-2 on open testing platforms under emergency use approval, namely, the COVIWOK COVID-19 RT-PCR Meril COVID-19 One-step RT-PCR Kit, the AmoyDx Novel Coronavirus (2019-nCoV) Detection Kit, the Meril COVID-19 One-step RT-PCR Kit and the NeoPlex COVID-19 Detection Kit, as alternatives to the current standard of care (SOC) assays in-country. All of the evaluated assays showed an acceptable performance, with a specificity of 100% and a sensitivity of 93.8% to 98.4%, compared to a SOC assay, with a Cohen’s kappa coefficient of ≥0.9 (95% CI). In addition, the assays detected the AccuPlex reference material at 100 copies/mL, suggesting a good limit of detection. These assays provide suitable alternatives to the SOC assays that are currently available in-country, and these alternatives are acceptable for diagnostic use in South Africa.

**IMPORTANCE** Laboratory diagnosis plays an important role in curbing the transmission of infection and reducing harmful delays in clinical and public health responses. Alternatives to the current standard of care assays for SARS-CoV-2 are important in order to overcome the challenges that are associated with global demands and supply shortages. Four molecular assays for the detection of SARS-CoV-2 that were designed for open testing platforms were evaluated in this study under emergency use approval. These assays had acceptable performance and provide suitable alternatives to the current standard of care assays that are available in-country. Their compatibilities with existing in-country amplification platforms make these assays convenient to use for diagnostic testing, both locally and globally These assays were recommended to the South African Health Products Regulatory Authority (SAHPRA) for patient care in South Africa.

## INTRODUCTION

Coronavirus disease 2019 (COVID-19), caused by severe acute respiratory syndrome coronavirus 2 (SARS-CoV-2), which emerged in late 2019, has been recognized as a pandemic by the World Health Organization (WHO) since March of 2020, and it has spread to almost every part of the globe (1, 2). As of June of 2022, over 500 million people have been infected worldwide, and more than 6 million deaths have been reported. In South Africa, the number of infections stands at approximately 4.0 million, with over 100,000 deaths having been reported so far (3).

The SARS-CoV-2 genome encodes both structural and nonstructural proteins. The most important structural proteins include the spike (S), envelope (E), and nucleocapsid (N) proteins that are encoded by genes that are located within the region just before the 3′ end of the genome (4). The RNA-dependent RNA polymerase (RdRp) protein is one of the 16 nonstructural proteins that is encoded by the open reading frame (*orf1ab*) gene, and it is known to be important for viral replication and transcription (5). Collectively, these have been recognized as important diagnostic targets for viral detection.

The enormous global burden caused by the pandemic led to the implementation of strict measures across the globe to control the infection and the spread of the virus. While the availability of SARS-CoV-2 vaccines and curative therapeutics have allowed for disease management to a certain degree (6), laboratory diagnostics nevertheless play an important role in the curbing of the transmission of infection and in the reduction of harmful delays in clinical and public health actions. Various laboratory testing approaches, including antigen and antibody tests, are currently available for diagnosing SARS-CoV-2 or for indicating exposure to SARS-CoV-2, respectively. However, molecular nucleic acid amplification tests that use real-time reverse transcription PCR (RT-PCR) technology remain the most sensitive diagnostic tool for the detection of SARS-CoV-2 and have been established as the gold standard for confirmatory diagnosis (7).

In South Africa, there are several SARS-CoV-2 RT-PCR diagnostic assays that are in use as the current standard of care (SOC) tests, and these have been approved by in-country and other regulatory authorities (8, 9). However, due to global demand and concerns of widespread kit shortages, especially during peaks of infection, it is important to evaluate and validate alternatives to these SOC assays in-country so that they would be readily available and provide reliable results in times of need.

The main aim of this study was to independently evaluate the analytical performance of four molecular RT-PCR kits for the detection of SARS-CoV-2. These standalone PCR assays are available in South Africa and include the COVIWOK COVID-19 RT-PCR Meril COVID-19 One-step RT-PCR Kit (SNP Biotechnology, Ankara, Turkey), AmoyDx Novel Coronavirus (2019-nCoV) Detection Kit (Amoy Diagnostics, Xiamen, China), Meril COVID-19 One-step RT-PCR Kit (Meril Diagnostics, Gujarat, India), and NeoPlex COVID-19 Detection Kit (GeneMatrix Inc, Gyeonggi-do, South Korea). These were investigated for use on open testing platforms to broaden access to testing, especially during peak testing schedules and with limited stocks of current closed testing platforms. A concise evaluation panel consisting of residual clinical specimens, commercially available reference materials, and viral cultures was used to evaluate the performance of these commercial kits (10).

## RESULTS

### Overall results.

All the RNA samples were processed, and the results were analyzed, according to the manufacturers’ recommendations, for the individual assays that were tested. Agreement data as well as cycle threshold (Ct) values for each gene target are provided in the Supplementary Table. For the Amoy assay, out of a total of 90 specimens and controls that were tested, 2 negative residual specimens amplify neither the target genes nor the internal control gene and were therefore considered to be invalid specimens (error rate of 2.22%; 2/90). A single residual positive specimen was falsely detected as a negative, and a single viral culture replicate at a 1:1,000,000 dilution was not detected. For the Meril assay, out of of a total of 90 specimens and controls tested, a single false-negative result was obtained, and the N gene target was not detected in 2 of the 3 replicates of the viral culture at a 1:1,000,000 dilution. For both the NeoPlex and COVIWOK assays, a total of 92 specimens and controls were tested. For the COVIWOK assay, neither gene target was detected in one viral culture replicate at 1:1,000,000 dilution. In addition, 3 false-negative specimens were identified. However, 2 of these were also negative on the confirmatory assay (TaqPath COVID-19 Multiplex Assay). Similarly, for the NeoPlex assay, 3 false-negative specimens were identified, and 2 replicates of the 1:1,000,0000 viral culture dilution were unable to detect either gene target. It is important to note that all of the false-negatives that were detected were from specimens that had a high Ct values (low viral content).

### Accuracy (sensitivity, specificity) and agreement (Cohen’s kappa coefficient).

**FIG 1:**
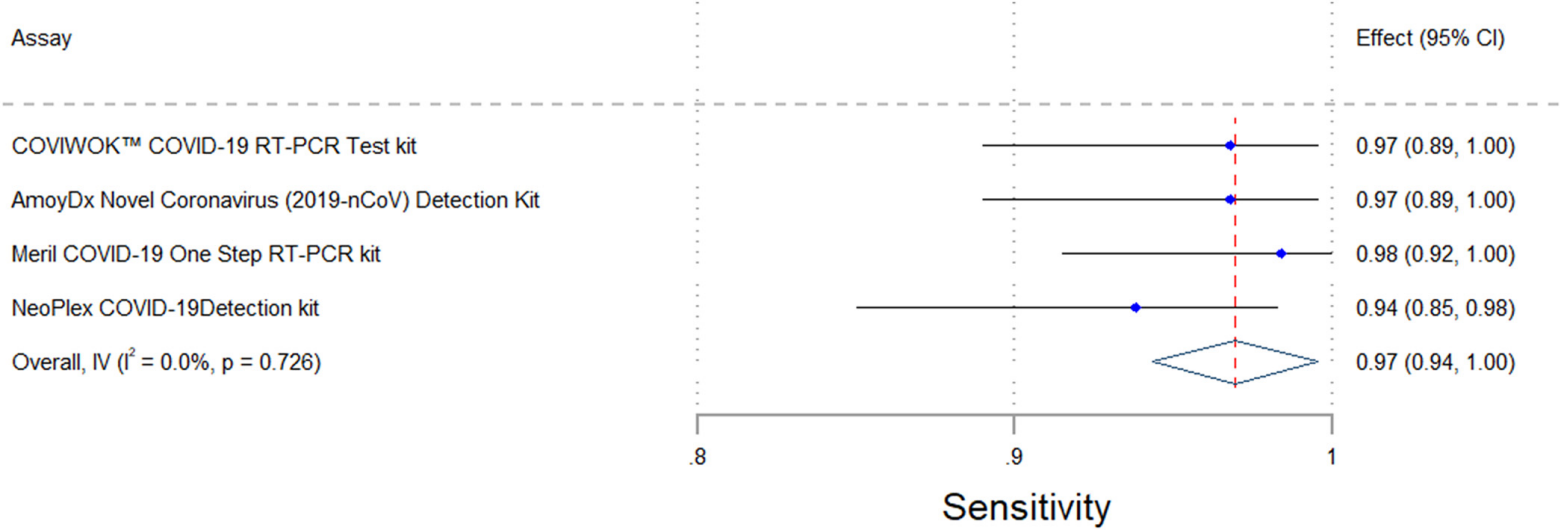
Forest plot showing the upper and lower confidence levels for the sensitivity of the four assays.

**FIG 2:**
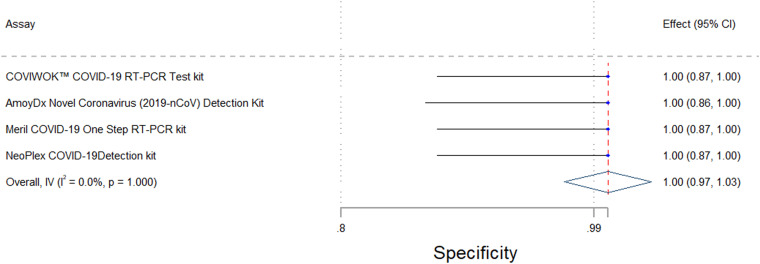
Forest plot showing the upper and lower confidence levels for the specificity of the four assays.

Accuracy was determined using clinically relevant specimens, kit positive and negative controls (*n* = 2), water blanks (*n* = 2), dilutions of live viral cultures (*n* = 12), diluted SeraCare (AccuPlex) samples (*n* = 14), ATCC reference material (*n* = 4), and IDT reference material (*n* = 2). For both the COVIWOK and Meril assays, 54 residual specimens were used. For the NeoPlex assay, 56 specimens were used. Two invalid specimens were excluded from the accuracy analysis for the Amoy assay. Therefore, only 52 clinical specimens were used. Table 1 as well as Fig. 1 and 2 provide a summary of the overall accuracy and agreement data for the four assays that were analyzed. All of the kits had a specificity of 100% and an acceptable sensitivity of ≥90% (9, 11). The Meril assay scored the highest sensitivity of above 98%, whereas the NeoPlex assay showed the lowest sensitivity of 94%. All of the assays had good agreement scores, with Cohen kappa coefficients of ≥0.9 (95% CI).

**TABLE 1:** Accuracy (sensitivity, specificity) and agreement (Cohen’s kappa coefficient) of the four assays^*a*^

Assay name	Type of specimens	Number of specimens	Sensitivity (95% CI)	Specificity (95% CI)	PPV (95% CI)	NPP (95% CI)	Cohen’s kappa (95% CI)	Agreement score
COVIWOK Covid-19 RT-PCR Meril COVID-19 One-step RT-PCR Kit	RNA	90	96.8% (89.0, 99.6)	100% (87.2, 100)	100% (94.1, 100)	93.1% (77.2, 99.2)	0.9482 (0.8773, 1.0191)	Very Good
AmoyDx Novel Coronavirus (2019-nCoV) Detection Kit	RNA	88	96.8% (89.0, 99.6)	100% (86.3, 100)	100% (94.1, 100)	92.6% (75.7, 99.1)	0.9454 (0.871, 1.0201)	Very Good
Meril COVID-19 One-step RT-PCR Kit	RNA	90	98.4% (91.5, 100)	100% (87.2, 100)	100% (94.2, 100)	96.4% (81.7, 99.9)	0.9738 (0.923, 1.025)	Very Good
NeoPlex Covid-19 Detection Kit	RNA	92	93.8% (85.0, 98.3)	100% (87.2, 100)	100% (94.1, 100)	87.1% (70.2, 96.4)	0.8995 (0.8037, 0.9953)	Very Good

a RNA, ribonucleic acid; PPV, positive predictive value; NPP, Negative predictive value; CI, confidence interval.

### Limit of detection (LOD).

The LOD was investigated using the AccuPlex reference control material (Table 2). Both the NeoPlex Kit (*RdRp* and *N*) and the Meril Kit (*ORF1ab* and *N*) detected the AccuPlex reference material for both genes at all of the dilutions and in all of the replicates that were tested. The Amoy assay also detected the reference material for both gene targets (*ORF1ab* and *N*) at 5,000, 1,000, 500, 250 and 100 copies/mL, but the *N* gene was detected in only 2 replicates at 100 copies/mL. The COVIWOK assay was able to detect the AccuPlex reference material for the *RdRp* gene at all of the dilutions and in all of the replicates that were tested (5,000, 1,000, 500, 250 and 100 copies/mL). However, for the *N* gene, the AccuPlex reference material was consistently detected only at 5,000, 1,000 and 500 copies, with variability at 250 copies/mL and no detection at 100 copies/mL.

**TABLE 2:** Limit of detection of the four assays assessed using the AccuPlex reference material^*a*^

Assay	Gene targets	5,000 copies/mL, *n* = 1	1,000 copies/mL, *n* = 3	500 copies/mL, *n* = 3	250 copies/mL, *n* = 3	100 copies/mL, *n* = 3
COVIWOK Covid-19 RT-PCR	*RdRp*	✓	✓✓✓	✓✓✓	✓✓✓	✓✓✓
Meril COVID-19 One-step RT-PCR Kit	*N*	✓	✓✓✓	✓✓✓	✓	-
AmoyDx Novel	*orf1ab*	✓	✓✓✓	✓✓✓	✓✓✓	✓✓✓
Coronavirus (2019-nCoV)	*N*	✓	✓✓✓	✓✓✓	✓✓✓	✓✓
Meril COVID-19 One-step RT-PCR Kit	*orf1ab*	✓	✓✓✓	✓✓✓	✓✓✓	✓✓✓
	*N*	✓	✓✓✓	✓✓✓	✓✓✓	✓✓✓
NeoPlex Covid-19	*RdRp*	✓	✓✓✓	✓✓✓	✓✓✓	✓✓✓
Detection Kit	*N*	✓	✓✓✓	✓✓✓	✓✓✓	✓✓✓

a The number of ticks under each dilution indicates the number of replicates detected.

### Precision analysis (reproducibility) and linearity.

Assay precision (standard deviation [SD] and percentage coefficient of variation [%CV]) and linearity were calculated using the reported Ct values of viral culture dilutions that were tested in triplicate (Table 3; Fig. S1). All of the kits that were analyzed showed acceptable precision (variability). The *N* and *orf1ab* gene targets in the Amoy assay were detected in 1:100,000 dilutions for all replicates. For the least concentrated viral culture dilution (1:1,000,000 dilution), the *orf1ab* gene target was not detected in any of the replicates, and the *N* gene was detected in two of the three replicates. Therefore, the %CV could not be calculated at this dilution. Similarly, for the Meril assay, the *N* gene was only detected in one replicate in the lowest viral culture dilution (1:1,000,000). Therefore, the %CV could not be calculated. However, the *orf1ab* gene target was detected in all of the culture supernatant dilutions that were tested. For both the COVIWOK assay and the NeoPlex Kit, good linearity was observed, with *R*^2^ values of 0.9758 and 0.9997, respectively, for the *RdRp* gene. A regression analysis could not be performed on the *N* gene due to insufficient data, as the viral culture dilutions of 1:100,000 and 1:1,000,000 produced negative results at the *N* gene target for the COVIWOK assay. For the NeoPlex assay, the viral culture at the 1:100,000 dilution produced negative results for the *N* gene, whereas variability was observed at the 1:1000,000 dilution for both gene targets.

**TABLE 3:** Precision overview of the four evaluated assays^*a*^

Assay	Gene targets	1:1,000 dilution			1:10,000 dilution			1:100,000 dilution			1:1,000,000 dilution			*R*^2^ value
		Mean Ct	SD	% CV	Mean Ct	SD	% CV	Mean Ct	SD	% CV	Mean Ct	SD	% CV	
COVIWOK Covid-19 RT-PCR	*RdRp*	31.1	0.3	0.9	33.9	0.3	0.8	38.8	0.4	1.0	Insufficient data			0.9758
Meril COVID-19 One-step RT-PCR Kit	*N*	31.7	0.2	0.8	34.6	0.5	1.4	Insufficient data						
AmoyDx Novel Coronavirus (2019-nCoV) Detection Kit	*orf1ab*	31.7	0.5	1.6	34.1	0.3	0.8	36.7	0.1	0.3	Insufficient data			0.9994
	*N*	31.3	0.2	0.7	33.6	0.3	1.1	36.6	0.1	0.3				0.9952
Meril COVID-19 One-step RT-PCR Kit	*orf1ab*	26.6	0.4	1.6	29.7	0.2	0.6	33.9	0.2	0.5	34.6	0.5	1.49	0.9364
	*N*	28.8	0.7	2.6	31.1	0.2	0.9	34.9	2.1	6.0	Insufficient data			0.9906
NeoPlex Covid-19	*RdRp*	30.0	0.9	3.2	33.4	0.3	1.0	37.2	0.1	0.2	Insufficient data			0.9997
Detection Kit	*N*	31.8	0.4	1.4	34.4	0.2	0.6	Insufficient data						

a Precision analysis was performed using live viral culture dilutions.

## DISCUSSION

Since the WHO declared COVID-19 a pandemic, great emphasis has been placed on the importance of molecular diagnosis for SARS CoV-2 to limit the spread of the virus and to appropriately treat those with serious infections. Accurate and timely laboratory testing and result output are important for decision-making, particularly during outbreaks, regarding the implementation of control strategies. The recommended test for the diagnosis of COVID-19 is RT-PCR. There are currently more than 500 conventional RT-PCR COVID-19 testing kits that are available commercially, including 64 assays that have been approved by the United States Food and Drug Administration (US-FDA EAU) and WHO-EUL (8). However, due to global demand and concerns of widespread, acute kit shortages, especially during peaks of infection, it has become important to evaluate and validate alternative kits that can be readily available in-country and can provide reliable results in times of need.

A number of studies have independently evaluated SARS-CoV-2 diagnostic kits with various performance outcomes (12–17). In this study, we evaluated the analytical performance of four standalone SARS-COV-2 RT-PCR kits that are available in South Africa, using a concise testing panel. The kits include the COVIWOK COVID-19 RT-PCR Meril COVID-19 One-step RT-PCR Kit, AmoyDx Novel Coronavirus (2019-nCoV) Detection Kit, Meril COVID-19 One-step RT-PCR Kit, and NeoPlex COVID-19 Detection kit. To the best of our knowledge, these kits and assays have not been evaluated previously. In this study, all four kits showed an acceptable performance, compared to a SOC test, namely, the Roche Cobas SARS-CoV-2 assay. The sensitivities ranged from 94% for the Neoplex assay to 98.4% for the Meril assay, and a specificity of 100% was observed for all of the assays that were investigated. The high level of agreement between the SOC and the assays is indicated by Cohen’s kappa values of ≥0.9.

All of the assays correctly identified all the negative residual clinical specimens that were tested, except the Amoy kit, which produced two invalid results. With regard to positive clinical specimens, the COVIWOK and the NeoPlex assays reported 3 false-negatives, whereas the Amoy and the Meril assays detected a single false-negative specimen. Considering that these were residual archived specimens with low viral loads (high Ct values), the integrity of the viral RNA that was present in some of these samples may have been compromised during the shipping and/or freeze-thawing, and this may have affected their detection. However, the overall capabilities of these kits to detect the presence of SARS-CoV-2 RNA are of importance.

Both the NeoPlex Kit and the Meril Kit detected the AccuPlex reference material for both genes at all of the dilutions and replicates tested, demonstrating an LOD of 100 copies/mL. This equates to 2 copies/reaction, considering that a total reaction volume of 20 μL was used for the assays. The LOD for the NeoPlex assay was not established by the manufacturer at the time of the study. However, an updated instructions for use manual indicates a LOD of 5.4 × 10^3^ NDU/mL (18). For the Meril assay, the LOD for only the *N* gene is provided by the manufacturer at <500 copies/reaction (19). Our study indicates a lower LOD for both assays, and we were additionally able to provide an LOD for both gene targets. Similarly, for the Amoy assay, the LOD in this study was determined to be 100 copies/mL, which is lower than the LOD of 500 copies/mL that was provided by the manufacturer (20). The LOD of the COVIWOK assay has been provided by the manufacturer to be 10 copies/reaction. This is in agreement with the results that were obtained in our study. The *RdRp* gene displayed an LOD of 100 copies/mL, whereas the *N* gene had an LOD of 500 copies/mL, which approximates to 2 copies/reaction and 10 copies/reaction, respectively (total reaction volume of 20 μL).

While all of the assays that were assessed showed acceptable overall precision for both genes, variability was mostly observed on the *N* gene target in higher dilutions (1:100,000 and 1:1,000,000). The reduced sensitivity that was observed may be attributed to the primer-probe sequence of the gene targets that was used in these assays. Nalla et al. (21) evaluated seven different primer-probe sets at various dilutions and found that the N2 set that was developed by the Centers for Disease Control and Prevention (CDC) (22) and the E-gene primer-probe set that was described by Corman et al. (23) to be the most sensitive. All of the sets were found to be highly specific. Therefore, it is important to assess the primer sequences before their inclusion into diagnostic assays. The presence of mutations on the N gene of SARS-CoV-2 may additionally impact the annealing of the primers and probes of RT-PCR diagnostic assays and may consequently affect performance. Lesbon et al. (24) sequenced SARS-CoV-2 genomes from 17 positive samples with an undetected N gene target from a RT-PCR assay. Three sets of mutations that affected the detection of the N gene were identified and were thought to be responsible for the reduced sensitivity that was observed. This may additionally contribute to false-negative results and affect the use of SARS-CoV2 real-time RT-PCR diagnostic kits, particularly those with a single gene target.

The specimens that were used in the testing panel were collected during a particular wave of infection when SARS-CoV-2 variants were not of a major concern. Therefore, further investigation is required to understand the compatibilities of these assays with emerging variants, as specimens selected from variant-driven waves of infection could affect the performance of the assays and could lead to false-negative results. Nevertheless, the evaluated assays target more than one SARS-CoV-2 gene. Therefore, a mutation in one gene may not affect the detection of infections.

In terms of their ease of use, all of the evaluated assays were easy to set up and perform. Clear instructions were provided in the package inserts. In addition, the presence of an endogenous internal control provides quality control specimen quality, extraction, and PCR. However, these assays should be performed by skilled laboratory personnel.

This study has some limitations. First, we were limited by the sample size that was used in this evaluation. Nevertheless, our data support that all of the assays had an acceptable performance. Second, the cross-reactivity of these assays in the presence of other human respiratory viruses was not tested. This could have an impact on the result outcome.

### Conclusion.

Considering the demand for reagents for SARS-CoV-2 RT-PCR diagnosis, especially during peak testing, it is necessary to independently assess and evaluate the performance of assays that are compatible with existing platforms and are readily available in-country to locally guarantee supplies. Based on their accuracy, all of the assays that were evaluated in this study had an acceptable performance and were recommended to the South African Health Products Regulatory Authority (SAHPRA) for patient care in South Africa. Furthermore, their compatibilities with existing in-country amplification platforms make these assays convenient to use for diagnostic testing. These kits provide a suitable alternative to current SOC testing, and their abilities to detect more than one gene target make these assays highly specific and robust, especially in light of emerging variants of concern, as a mutation in one gene target will not affect detection in the second target.

## MATERIALS AND METHODS

Sample processing and specimen handling were done following strict laboratory safety protocols. A biosafety level 3 laboratory and biohazard cabinets were used for all sample processing. Personal protective equipment, including masks, gowns, goggles, and gloves were used throughout the conduct of the study and for all other lab-related procedures.

### PCR assays and testing platforms.

Table 4 provides details of the assays that were evaluated and of the testing platforms that were used in this study. Downstream RNA processing, including master mix preparation, cycling protocols, and result interpretation, were carried out in accordance with the manufacturers’ instructions. These kits collectively amplified and detected three distinct gene targets: *orf1ab*, *N*, and *RdRp* of SARS-CoV-2.

**TABLE 4:** Details of the four evaluated kits and the amplification platforms used^*a*^

Assay name	Company	Country	South African supplier	Regulatory status	Gene targets	Internal control gene	RNA template vol (μL)	Total reaction vol (μL)	Thermocycler used	Manufacturer recommended Ct cutoff
COVIWOK Covid-19 RT-PCR Meril COVID-19 One-step RT-PCR Kit	SNP Biotechnology	Turkey	LASEC Group	CE-IVD, ICMR	*RdRp* and *N*	Yes	10	20	CFX96 Real-time PCR Machine (Bio-Rad Laboratories, Hercules, CA),	≤35 (IC); ≤43 (gene targets)
AmoyDx Novel Coronavirus (2019-nCoV) Detection Kit	Amoy Diagnostics	China	Celtic diagnostics/LASEC Group	CE-IVD	*orf1ab* and *N*	Yes	9	40	QuantStudio 5 System (Thermofisher Scientific)	>45 (NTC); <32 (PC); ≤40 (IC); ≤37 (gene targets)
Meril COVID-19 One-step RT-PCR Kit	Meril Diagnostics	India	Meril SA	CE-IVD	*orf1ab* and *N*	Yes	10	20	QuantStudio 5 System (Thermofisher Scientific)	>40 (NTC); ≤35 (PC); ≤40 (IC); ≤40 (gene targets)
NeoPlex Covid-19 Detection Kit	Genematrix	Korea	Abafazi Healthcare	US FDA (EAU); CE-IVD	*RdRp* and *N*	Yes	5	20	CFX96 Real-time PCR Machine (Bio-Rad Laboratories, Hercules, CA, USA)	≤38

a CE-IVD, European Conformity-In Vitro Diagnostic; COVID-19, coronavirus disease 2019; NTC, no template control; PC, positive control; IC, internal control; *N*, nucleocapsid gene; *orf1ab*, an open reading frame; *RdRp*, RNA-dependent RNA polymerase gene.

### Primary residual clinical specimen swabs.

Ethics approval was obtained from the University of the Witwatersrand Human Research Ethics Committee under M1911201 to access residual clinical specimens, post-routine testing for patient management. Residual clinical specimen swabs in phosphate-buffered saline (PBS) (Gibco, Life Technologies, The Netherlands), universal transport medium (UTM), or viral transport medium (VTM) that were previously collected for routine SARS-CoV-2 testing were stored at −80°C until needed. These specimens were collected from patients who visited health care centers, mainly during the first COVID-19 infection wave in the country. The collected specimens were processed and tested on the SOC assay immediately as they arrived at a routine testing facility.

The specimens were thawed, and RNA was extracted using the Tianlong Nucleic Acid Extraction Kit (T014H) and the Tianlong Nucleic Acid Extraction platform (Tianlong Technology Co. Ltd., Xi’an China). Clinical specimens were selected across a range of cycle threshold (Ct) values as a proxy measure of viral load (VL) (25), based on the comparator method that was used for initial testing (Roche cobas SARS-CoV-2 assay; Roche Molecular, Pleasanton, CA, USA): Ct = 17.4 to 35.8/36.3 (median Ct = 25.70/26.50) for the *orf1ab* gene, and Ct = 18.1 to 38.40 (median Ct = 26.50) for the *E* gene. While the same specimen panel was used throughout the evaluation process, insufficient extracted RNA from two residual clinical specimens resulted in a difference in the number of clinical specimens used: *n* = 56 (for the NeoPlex and COVIWOK assays) and *n* = 54 (for the Amoy and Meril assays). The panel included specimens reporting the presence (*n* = 34 for Amoy and Meril assays and *n* = 36 for NeoPlex and COVIWOK assays) or absence (*n* = 20) of SARS-CoV-2, as reported by the routine patient management testing.

### SARS-CoV-2 viral culture supernatants.

The viral culture supernatants consisted of known concentrations of four different dilutions, tested in triplicate (*n* = 12). SARS-CoV-2 viral cultures were originally obtained through collaboration with Wolfgang Preiser (Department of Medical Virology, Stellenbosch University, Stellenbosch, Western Cape, South Africa) and Bavesh Kana (CBTBR, University of the Witwatersrand, Johannesburg, Gauteng, South Africa). The concentration of the original viral culture was calculated using semiquantitative PCR in the lab, and it was estimated to be 1 × 10^5^ copies/μL. 4 viral culture supernatant dilutions in PBS were prepared in a biological safety laboratory level 3, and these included 1:1000, 1:10,000, 1:100,000 and 1:1,000,000 (which approximates log 5.0, log 4.4, log 3.4 and log 2.5 viral copies per milliliter [cp/mL], respectively). These were prepared in 15 mL Falcon tubes (Thermo Fisher Scientific). Nest Biotechnology oropharyngeal specimen collection swabs (Whitehead Scientific Pty., Ltd.) were used to prepare swabs of the different dilutions via swab capture. Briefly, place and swirl the swabs individually in each dilution for 30 s (15 s clockwise and 15 s counterclockwise). Place the swab into a labeled cryovial, snap at the breakpoint, and seal the cryovial. Add 1 mL of PBS to each swab in the existing cryovial. Seal and vortex vigorously for 1 min. Leave at ambient temperature for 10 min before testing on a standard of care test, prior to inclusion in the evaluations. RNA was extracted as described above.

### Reference material.

Undiluted and diluted SARS-CoV-2 whole-genome positive and negative controls (AccuPlex; LGC SeraCare, Milford, MA, USA) were included in the evaluation panel. Specifically, these consisted of an undiluted negative AccuPlex control, an undiluted AccuPlex positive control (5,000 cp/mL) (both of which were tested once), and four positive-control dilutions (1,000 cp/mL, 500 cp/mL, 250 cp/mL and 100 cp/mL) (all tested in triplicate [*n* = 14]). RNA was extracted as described above, using a Tianlong Nucleic Acid Extraction Kit and a Tianlong Extraction platform. Additional reference material included synthetic RNA from the American Tissue Culture Collection (ATCC) (*n* = 4) and plasmid controls from Integrated DNA Technologies (IDT; Coralville, IA, USA), (*n* = 2). The ATCC material consisted of ATCC-VR-1986D (genomic RNA that was isolated from a preparation of severe acute respiratory syndrome-related coronavirus 2 strain 2019-nCoV/USA-WA1/2020), ATCC-VR-3262D (Synthetic Human coronavirus HKU1 RNA), ATCC-VR-3276D (preparation includes fragments from the ORF1ab, envelope, and nucleocapsid regions), and ATCC-VR-1558T (RNA from Betacoronavirus 1 OC43). The IDT plasmids included a negative control and a positive control.

### Comparator assay and statistical analysis.

The cobas SARS-CoV-2 assay (Roche Molecular, Pleasanton, CA, USA) was used as the comparator. The results obtained via the evaluated kits were compared to those obtained via the comparator assay. The statistical analysis, including the accuracy (sensitivity and specificity) and percentage agreement analyses (Cohen’s kappa coefficient) was carried out using Stata version 14 (StataCorp, College Station, TX, USA). To ensure that specimen quality was not compromised, RNA extracts were tested on a confirmatory assay (Applied Biosystems TaqPath COVID-19 CE-IVD RT-PCR Kit [TaqPath], Waltham, MA, USA).
