# Supplementary material for: Performance Evaluation of Four Qualitative RT-PCR Assays for the Detection of Severe Acute Respiratory Syndrome Coronavirus 2 (SARS-CoV-2)
Source: Microbiol Spectr. 2023 Feb 28;11(2):e03716-22. doi: 10.1128/spectrum.03716-22 (PMC10101067; doi:10.1128/spectrum.03716-22)
Supplement: Supplemental file 1 — Fig. S1. Download spectrum.03716-22-s0001.pdf, PDF file, 0.4 MB [file spectrum.03716-22-s0001.pdf]

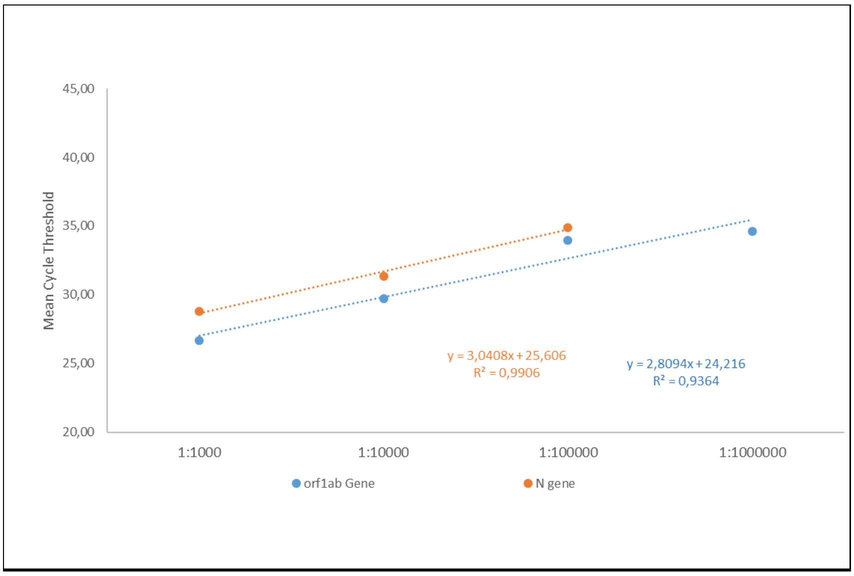
45,00
40,00
35,00
30,00
25,00
20,00
Mean Cycle Threshold
y = 3,0408x + 25,606
R² = 0,9906
y = 2,8094x + 24,216
R² = 0,9364
1:1000
1:10000
1:100000
1:1000000
orf1ab Gene
N gene

A

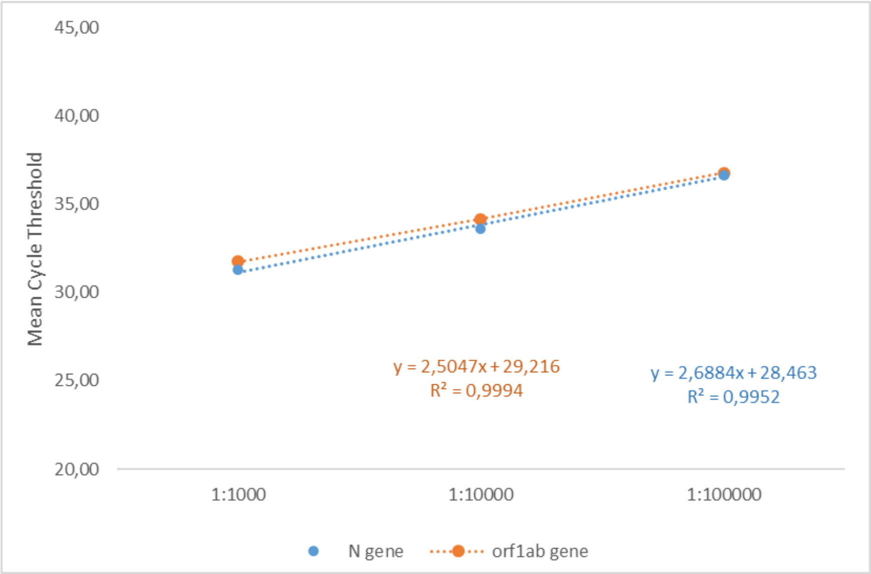
45,00
40,00
35,00
30,00
25,00
20,00
Mean Cycle Threshold
y = 2,5047x + 29,216
R² = 0,9994
y = 2,6884x + 28,463
R² = 0,9952
1:1000
1:10000
1:100000
N gene
orf1ab gene

B

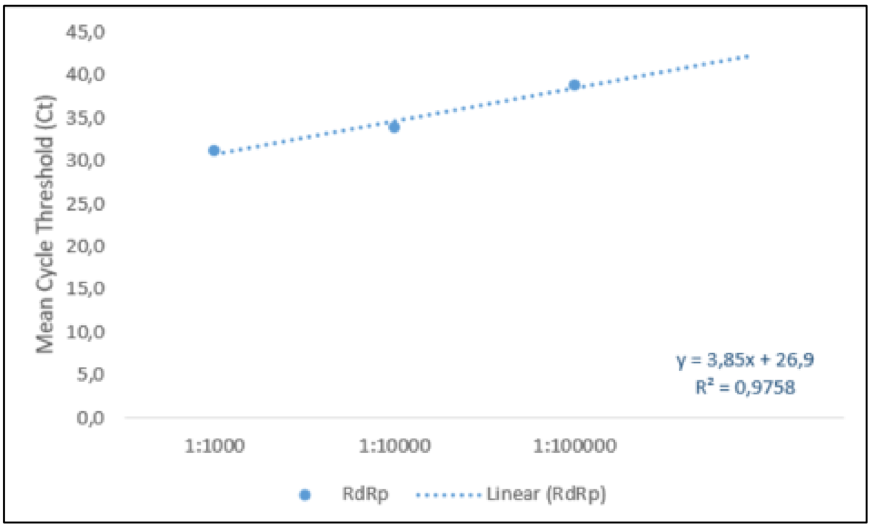
45,0
40,0
35,0
30,0
25,0
20,0
15,0
10,0
5,0
0,0
Mean Cycle Threshold (Ct)
y = 3,85x + 26,9
R² = 0,9758
1:1000
1:10000
1:100000
RdRp
Linear (RdRp)

C

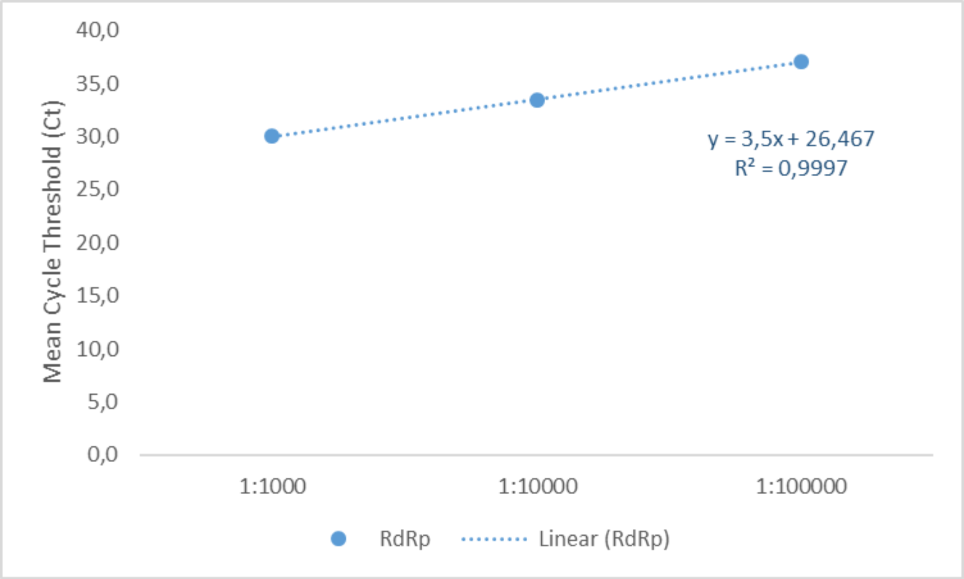

D

Supplementary Figure 1: Linear regression of mean Ct (triplicate) of viral culture lysates tested across a range of dilutions using the (A) Meril assay, (B) Amoy assay, (C) Coviwok assay and (D) NeoPlex assay from RNA extracted using the Tianlong Nucleic Acid Extraction Kit (T014H) and the Tianlong Nucleic Acid Extraction platform. Equations of the line are represented in the graph. Linearity was not performed for the N gene for both the Coviwok and the Neoplex assays due to insufficient data.
